# The Relation of Environment to Unipolar Recurrent Depression

**DOI:** 10.1192/j.eurpsy.2022.757

**Published:** 2022-09-01

**Authors:** N. Horesh Reinman

**Affiliations:** Bar Ilan university, Psychology, Ramat Gan, Israel

**Keywords:** life events, depression, recurrent mood disorder

## Abstract

**Introduction:**

Recurrent Unipolar and Bipolar affective disorders are considered paradigms of biological entities in psychiatry. However recent theories have underlined the role that environment plays in the genesis of these disorders in interaction with genetic diatheses.

**Objectives:**

This study examined the relationship between stressful life events (SLE) and recurrent major depressive disorders.

**Methods:**

Three groups of 50 subjects were assessed: Patients with recurrent major depressive disorder with melancholic features; patients with borderline personality disorder; and healthy controls. Interviews for DSM-V Disorders were used for diagnosis. Beck Depression Inventory, The Israel Psychiatric Research Interview Life Event Scale and the Coddington Events Schedule were used to measure life events and depression and were confirmed with an interview.

**Results:**

The proportions of loss-related events in childhood and in the year preceding the first episode was higher in the depressed group than in the control groups during the same time period. Proportions of SLE, uncontrolled and independent events were also more common in the depressed patients in the year preceding the first episode.

**Conclusions:**

The study’s conclusion is that SLE plays an important role in the onset of depressive disorders. There are specific kinds of SLE that occur in childhood and in the year preceding the first episode. SLE has a less significant role in the maintenance of this illness.

**Disclosure:**

No significant relationships.

